# Associations between poverty, mental health and substance use, gender power, and intimate partner violence amongst young (18-30) women and men in urban informal settlements in South Africa: A cross-sectional study and structural equation model

**DOI:** 10.1371/journal.pone.0204956

**Published:** 2018-10-03

**Authors:** Andrew Gibbs, Rachel Jewkes, Samantha Willan, Laura Washington

**Affiliations:** 1 Health Economics and HIV/AIDS Research Division (HEARD), University of KwaZulu-Natal, Durban, South Africa; 2 Gender and Health Research Unit, South African Medical Research Council, Pretoria, South Africa; 3 Project Empower, Durban, South Africa; TNO, NETHERLANDS

## Abstract

Research suggests that poverty is a key driver of intimate partner violence (IPV), however detailed analysis suggests that this relationship is not clear, either for women’s experience or men’s perpetration of IPV. We explored associations between poverty and IPV using cross-sectional data from the Stepping Stones and Creating Futures cluster randomized control trial, in urban informal settlements in Durban, South Africa, with young (18–30) people. Using logistic regression and structural equation modelling we assess associations between poverty and women’s experience and men’s perpetration of physical and/or sexual IPV in the past 12 months. 680 women and 677 men were recruited into the study between September 2015 and September 2016. The analyses highlight how specific forms or measures of poverty intersecting with gender identities shape IPV. For men we found indicators of economic provision were associated with IPV perpetration, while for women food-insecurity was key to IPV experience. We also found similarities between women and men. First, food-insecurity and childhood traumas shaped pathways to substance misuse and poor mental health that increased IPV. Second, there was a resilience pathway in both models, whereby those with more education had increased gender equitable attitudes and fewer controlling behaviours, which reduced IPV. Interventions to reduce IPV need to work to reduce household food insecurity, but these need to be combined with gender transformative interventions. Interventions should also focus on reducing the impact of mental health and substance misuse. Finally, working to increase educational attainment is a long-term critical intervention to reduce IPV.

**Trial registration:** NCT03022370. Registered 13 January 2017, retrospectively registered.

## Introduction

While women’s experience of intimate partner violence (IPV) is common globally across all economic strata, with an estimated 30% of women experiencing physical and/or sexual IPV in their lifetime [1], there is a broad assumption that poverty is a key driver of IPV [2, 3]. Yet, there are multiple ways of assessing poverty, at the national, household, and individual level, and each of these provides different understandings of the relationships between poverty and IPV.

For women, at the household level there are relatively consistent associations between household food-insecurity and IPV, whereby higher levels of food insecurity increase women’s experiences of IPV [4, 5], even when controlling for overall economic status. In a multi-country study of women’s experience of IPV in Asia-Pacific (Cambodia, China, Papua New Guinea and Sri Lanka) food-insecurity at the household level was associated with increased experience of IPV [6]. And the association between food-insecurity and IPV also holds true in the global north [5]. In addition, studies on household socio-economic status (SES), have also broadly suggested that living in a household with a higher SES is associated with reduced recent IPV [7]. It is suggested that the association between household food insecurity and IPV, is linked to increased levels of stress and conflict in such households over the lack of resources, as well as women’s increased economic dependency on men, making it harder to leave abusive relationships [2, 8].

In contrast, studies on household assets and their association to IPV have found mixed results [9]. Most recently an analysis of 28 Demographic Health Surveillance (DHS) studies found that in three countries women’s ownership of assets were protective of IPV, in five countries women’s ownership of assets increased IPV, and in the others had no impact on IPV [10]. However, in Vietnam a study showed that it was women’s ability to assert control over assets (i.e decide on an assets use, rather than necessarily ownership) that made assets protective for IPV [11].

At the individual level, measures of poverty also have complicated associations with IPV. For instance, women’s engagement in paid work, while broadly empowering for women, has mixed associations with their experience of IPV [9]. In one global study, while work was broadly protective of IPV for women, where women’s involvement in paid work was not common, work increased women’s experience of IPV [12].

The evidence on the association between poverty and men’s perpetration of IPV is also unclear. Qualitative research has developed a strong argument that when men are economically marginalized from the capitalist economy, and unable to achieve markers of ‘masculinity’ and ‘respect’ through economic provision in relationships, they may establish an alternative masculinity, which uses violence and control over women as a resource for masculinity [13, 14]. Quantitative studies show a complicated picture in relation to this. In a multi-country study in Asia-Pacific, household food insecurity was associated with sexual IPV perpetration, and physical and sexual IPV perpetration in two out of six countries, but with no association in the other four countries [15].

The association between work and male perpetration of IPV is also mixed. In a young (15–19) population in urban South Africa, young men’s employment was protective of IPV-perpetration [16], while in the IMAGES multi-country study men’s employment had no association with their lifetime perpetration of physical IPV [17]. While a population based study in urban South Africa [18] showed working in the past 12 months increased men’s perpetration of IPV. As such, there appears to be no clear associations between men’s work and IPV perpetration.

Studies on rape perpetration (including non-partner rape) also highlight complicated associations between poverty and rape. In a multi-country study from Asia-Pacific, non-partner rape perpetration was associated with current food insecurity [19]. Yet a series of studies on rape perpetration from South Africa, showed men who were slightly better off than their peers–although living in overall contexts of poverty–were more likely to rape [20]. In these studies, slightly better off, was defined as their mother having a high school education.

There is also a complex relationship between poverty, mental health, and IPV for women and men. Poverty is a clear driver of poor mental health and substance misuse, including problematic alcohol use and depression [21–23]. Additionally, for women who experience IPV, depression, alcohol use, and PTSD are often co-morbid and consequences of IPV [24–26]. Moreover, depression, alcohol use and PTSD are also increasingly recognized as drivers of IPV perpetration by men, and experience by women [24]. A study in South Africa, [27] showed that for women PTSD, alcohol abuse and depression all mediated pathways between childhood abuse and experience of IPV, and that these clustered together. Similarly, amongst men in South Africa, PTSD mediated the pathway between childhood traumas and IPV perpetration [18].

Globally urban informal settlements have been growing rapidly. In sub-Saharan Africa, the urban population is expected to double in the next two decades [28], and two-thirds of these urban dwellers live in informal settlements [28]. Since the 1980s, South African informal settlements have been growing despite government attempts to provide subsidized housing [29]. Urban informal settlements have multiple health challenges, including high levels of HIV and IPV [28, 29].

The objective of this paper is two-fold. First to understand the relationship between poverty and women’s experiences, and men’s perpetration, of physical and/or sexual IPV. And second, to understand the potential pathways between these, specifically whether alcohol, mental health, and/or gender attitudes and practices, mediate these.

## Materials and methods

### Setting

The study was conducted in urban informal settlements in eThekwini Municipality, on the east coast of South Africa, between September 2015 and September 2016. eThekwini Municipality is the third largest city in South Africa, with just over half a million residents. There are an estimated 500 informal settlements, comprising about a quarter of the population of eThekwini Municipality [30].

### Research design

A non-representative cross-sectional study, forming the baseline of the Stepping Stones and Creating Futures (SS/CF) intervention trial, a cluster randomized control trial. The SS/CF trial is evaluating whether a combined livelihoods strengthening and gender transformative intervention amongst young (18–30) women and men can reduce men’s perpetration and women’s experience of IPV over a two-year period [31]. More information on the trial design has been published elsewhere [31].

### Ethics

The South African Medical Research Council and the Biomedical Research Ethics Committee at University of KwaZulu-Natal provided ethics approval. Community leaders provided letters of support. Participants signed informed consents in English, isiZulu or Xhosa. Participants in the control arm received R300, and in the intervention arm R100, as incentive for questionnaire completion. The differential amount reflecting challenges in retaining the control arm over a two-year period [31].

### Procedures

In mid-2015 thirty-four clusters of urban informal settlements in eThekwini Municipality were recruited into the study and randomized into control and intervention arms. Between October 2015 and September 2016 clusters were approached and community meetings held to explain the study. In each cluster approximately 20 women and 20 men were recruited through purposive and snow-ball sampling by a community-based organization, Project Empower, and screened for eligibility. Eligibility requirements were that participants were aged 18–30, had to be resident in the informal settlement, not in formal employment, and were able to complete informed consent. At recruitment, participants were not blinded to study arm [31]. Because of the sampling design (purposive and snow-ball), there is no information on refusal rates, and the sample size was based on trial outcome estimates [31], where we sought to recruit 680 men, and 680 women into the study.

Participants self-completed questionnaires on cellphones in English, isiZulu or Xhosa, in the community where they resided. Cellphones had pre-programmed questionnaires (with women’s and men’s separate) with logical skip patterns built-in. Same-sex fieldworkers were available to support participants if literacy was an issue. Completion of questionnaires took between 45 minutes and 1.5 hours.

### Measures

**Fig 1:**
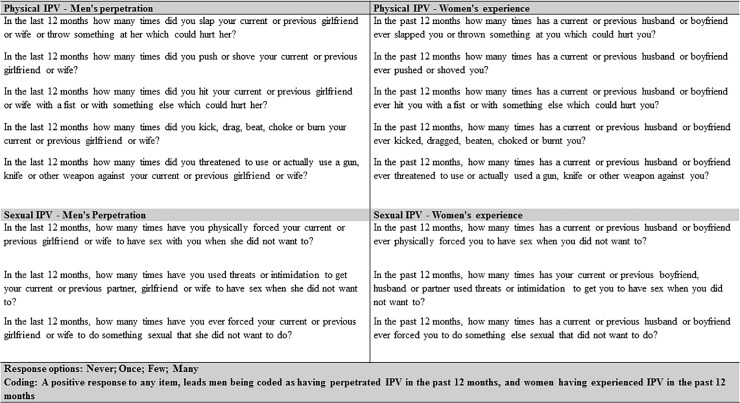
Definition of intimate partner violence.

The primary outcome for this analysis was women’s experience, and men’s perpetration, of physical and/or sexual IPV in the past 12 months. This was assessed through a modified WHO Violence Against Women (VAW) scale previously adapted for South Africa [32]. Five behaviourally specific questions asked about physical IPV and three about sexual IPV, in the past 12 months (Fig 1). Participants could respond never, once, few, many. Anyone responding positively to any item once, or more, was classified as experiencing (women) and perpetrating (men) IPV in the past 12 months.

As women experience the overwhelming majority of IPV, and men are the primary perpetrators of this [33], we only asked about these categorisations. In addition, we combined physical and/or sexual IPV into one measure, following the approach used in multi-country studies on factors associated with physical and/or sexual IPV [34], and refer to this as IPV moving forwards.

#### Covariates

Participants were asked about demographic characteristics. Specifically, age, education level–including whether they had a secondary school leaving certificate—and whether they had a partner who they were cohabiting with, or not. We included those without a current main partner, because the overwhelming majority of young people reported a primary partner in the past year and the outcome was in the past year. In addition, those without a main partner would lead towards a null result in associations, and as such this is a conservative modelling strategy.

Poverty and livelihoods were assessed through eight measures (Table 1). Household poverty was assessed through food-insecurity via the Household Hunger Scale, and recoded as recommended into a three-level variable (none or little, moderate, high) [35]. Five measures assessed work, and the impact of lack of work, on participants. Participants were asked about earnings in the past month, which was recoded into none versus any. Work consistency in the past year was a single item with participants classified into either working each month or most months, or less than this. Stress about lack of work (Cronbach α = 0.75 women; α = 0.78 men), and shame about lack of work (Cronbach α = 0.60 women, α = 0.57 men), were both assessed with four-items, with higher scores indicating greater stress, and greater shame respectively. Both scales had previously been used in South Africa [36]. Livelihood efforts were assessed with seven items asking about attempts to find work in the past three months (Cronbach α = 0.75 women; α = 0.74) with higher scores indicating more effort. Finally, two other items assessed individual level strategies of survival. One item asked whether participants had stolen in the past month because of hunger, this was coded as never or ever. Borrowing in the past month because of hunger, was coded weekly or more or less than weekly.

**Table 1:** Measures of poverty used in the study.

Measure	Item(s)	Response	Coding
Household food insecurity	In the past 4 weeks, how often was there no food to eat of any kind in your house because of a lack of money? In the past 4 weeks how often did you or any member of your household go to sleep hungry because of lack of food? In the past 4 weeks how often did you or any of your household go a whole day and night without eating because of lack of food?	Never, rarely, sometimes, often	Never or little; moderate; high
**Work and impacts of lack of work**			
Earnings in the past month	Considering all the money you earned from jobs or selling things, how much did you earn last month?	Number	None versus any
Work consistency	In the past 12 months how often did you work? Each month, most months, once in a while, never worked	Each month; most months; once in a while; never worked	Each month/most months versus once in a while/never worked
Stress about lack of work	a) I am frequently stressed or depressed because of not having enough work b) I am frequently stressed or depressed because of not having enough income c) I am frequently stressed or depressed because I am not proud of what I do to get money d) I am frequently stressed or depressed because I want or have to help my family with money	Strongly disagree; disagree; agree; strongly agree	Summed and higher scores indicates greater stress
Shame about lack of work	a) I sometimes feel ashamed to face my family because I am out of work. b) I spend most of my time out of work or looking for work c) I have given up looking for work because I never find any d) I am ashamed to see my girlfriend because I don’t have money	Strongly disagree; disagree; agree; strongly agree	Summed and higher scores indicates greater shame
Livelihood efforts	For the following statements, please tell me how often in the last 3 months you have done the following: a) Searched in town for work b) Searched newspapers for jobs c) Handed in or sent off an application for work d) Offered to work without pay to get experience e) Worked without pay to get experience f) Developed an idea for a way of earning by selling or making things g) Earned money through selling or making things	Never; once; a few times; many times	Summed and higher scores indicates more livelihood efforts
**Individual level strategies**			
Stolen in the past month	How often in the past month have you taken something that was not yours because you did not have enough food or money?	Never; once; two or three times; four or more times	Never versus once or more
Borrowed in the past month	How often in the past month have you had to borrow food or money because you did not have enough?	Every day; almost every week; once or twice; never	Never/once or twice versus everyday/almost every week

Gender attitudes were assessed with 20 items based on the Gender Equitable Men’s Scale modified for South Africa (Cronbach α = 0.86 women; α = 0.86 men). Higher scores indicated less equitable attitudes. Relationship control was assessed using a modified sexual relationship power scale (Cronbach α = 0.88 women; α = 0.87 men), with higher scores indicating more controlling behaviours [37]. A single item asked about quarrelling in the relationship, and was treated as a continuous score.

Childhood trauma was assessed using an adapted childhood trauma scale, comprising of 12 items, previously used in South Africa [32]. Participants were asked about experiences before the age of 18, around emotional and physical neglect, and sexual and physical abuse (Cronbach α = 0.82 women; α = 0.86 men).

Mental health and substance use were assessed with eight separate scales. Alcohol use was assessed with the 20-item AUDIT scale (Cronbach α = 0.81 women, α = 0.79 men) and treated as a sum-score, with higher scores indicating higher alcohol use. A single item asked about drug use in the past year and had a binary response. Depression symptomology was assessed with the Centre for Epidemiological Studies Depression (CESD) scale, with 20 items (Cronbach α = 0.88 women, α = 0.87 men) [38]. Post-traumatic stress disorder (PTSD) symptoms were assessed with the Harvard Trauma Questionnaire, with 16 items, asking about symptomology of PTSD (Cronbach α = 0.92 women; α = 0.92 men) [39]. Hope was assessed using six items of the Snyder Hope scale (Cronbach α = 0.82 women; α = 0.82 men). Questions on hope included, “There are lots of ways around any problem”. Four questions asked about satisfaction with life, based on the Satisfaction With Life Scale (Cronbach α = 0.67 women; α = 0.68) and higher scores indicated greater satisfaction [40]. A single item asked about how successful they felt in comparison to other women (men) your age, higher scores indicated greater life success.

### Analysis

Analysis was conducted in STATA14/IC. Analyses were at the individual level, took into account the structure of the data, and adjusted for clustering. Men and women were analysed separately.

Descriptive statistics were first estimated comparing the primary outcome for men (physical and/or sexual IPV perpetration) and women (experiencing physical and/or sexual IPV) against all covariates. Pearsons Chi-Squared assessed binary outcomes, while t-tests reporting p-values assessed differences between continuous variables. Variables were selected for inclusion based on theoretical assumptions about the causes of IPV from literature.

We built Gaussian random effects logistic regression models, at the individual level, accounting for data clustering to assess associations between covariates and men’s perpetration, and women’s experience, of IPV. We inputted any variable significant in descriptive statistics at the p<0.1 level, and controlled for age, education level and intervention arm. We conducted backwards elimination of variables, consecutively removing variables not making statistically significant contributions to the model. We then re-estimated the model. Backwards elimination is less likely to encounter Type II errors [41]. We continued the process until all variables were significant (p<0.05). Because of the large number of outcomes in this population, we had adequate sample for the number of explanatory variables, based on a rule of thumb of 10 outcomes per explanatory variable [42].

We conducted Structural Equation Modelling (SEM) with maximum likelihood estimation to test structural pathways between poverty and IPV, and the potential role of mediating variables selected from significance in the regression model and theoretically considered. Initially we constructed and tested a latent variable for poverty based on the household food-insecurity questions. We fitted the structural model and removed pathways that were non-significant at the alpha <0.05 level and removed variables observed and latent that did not contribute to any pathways towards IPV experience (women) and perpetration (men). We assessed covariance and added in additional covariance between observed variables. We tested for women and men the directionality of the household food insecurity-childhood trauma relationship, and show the models with the best fit. In terms of contemporaneous nature of food-insecurity/childhood trauma, following Jewkes et al. (2017) we assume that there is little change in food security overtime, this is particularly the case in the South African context, given the worsening state of the South African economy and little change in overall food-insecurity in the country. Both models showed satisfactory goodness of fit: for women RMSEA = 0.017, CFI = 0.995 and TLI = 0.992, and for men RMSEA = 0.025, CFI = 0.988 and TLI = 0.981, before adjustment for clustering.

## Results

In total we recruited 680 women and 677 men. Two-thirds (65.2% 95%CI:61.5–68.7%) of women reported experiencing any physical and/or sexual IPV in the past year, while just over half of men (56.9%, 95%CI:53.1–60.6%) of men reported perpetrating physical and/or sexual IPV in the past year. In bivariate analysis (Table 2) a lower proportion of men reporting having currently not having a partner perpetrated IPV (p = 0.0004). While a larger proportion of women who lived with their partner reported experiencing IPV (p<0.0001). For women, a smaller proportion of those who completed secondary school reported past year IPV experience (p = 0.009).

**Table 2:** Descriptive associations between men's perpetration of, and women's experience, of physical and/or sexual IPV and socio-demographics, livelihoods, gender, and mental health (N = 677 men; 680 women).

			Men					Women		
			No IPV perpetrated past 12m	IPV perpetrated past 12m				No IPV experienced in past 12m	IPV experienced in past 12m	
Socio-demographics	N	n	%/mean (95%CI)	%/mean (95%CI)	p-value	N	n	%/mean (95%CI)	%/mean (95%CI)	p-value
Age		677	23.68(23.29–24.08)	23.56(23.24–23.88)	0.628		680	23.41(23.00–23.83)	23.80(23.49–24.12)	0.143
Education	677					680				
Only primary		77	11.00(7.88–15.14)	11.46(8.66–15.02)			55	6.33(3.84–10.25)	9.03(6.70–12.06)	
Secondary, but not completed		393	53.61(47.91–59.22)	61.46(56.49–66.20)			419	56.12(49.77–62.27)	64.56(59.97–68.90)	
Completed secondary		207	35.40(30.18–40.98)	27.08(22.93–31.68)	0.064		206	37.55(31.70–43.80)	26.41(22.52–30.70)	0.009
Relationship status	677					680				
Living with partner		73	7.90(5.32–11.59)	12.76(9.80–16.46)			113	10.97(7.57–15.64)	19.64(16.26–23.52)	
Partner, but not living with		459	63.92(58.21–69.26)	70.83(66.08–75.17)			441	71.31(65.22–76.71)	61.40(56.81–65.79)	
No current partner		145	28.18(23.26–33.68)	16.41(13.04–20.44)	0.001		126	17.72(13.42–23.03)	18.96(15.59–22.86)	0.009
**Livelihoods**										
Household food insecurity	676					680				
None or little		125	21.03(16.74–26.09)	16.43(13.06–20.40)			127	27.43(22.16–33.41)	14.00(11.06–17.56)	
Moderate		382	57.24(51.49–62.80)	56.25(51.24–61.14)			342	48.10(41.82–54.45)	51.47(46.82–56.09)	
High		169	21.72(17.33–26.87)	27.34(23.08–32.06)	0.134		211	24.47(19.47–30.28)	34.54(30.24–39.10)	<0.001
Earnt any money in past month (> = R1; yes)	676	416	52.76(47.04–58.41)	68.23(63.32–72.77)	<0.001	680	205	31.22(25.62–37.43)	29.57(25.49–34.01)	0.656
Worked in the past 12months (most or each month)	676	157	16.21(12.35–20.98)	28.39(24.13–33.06)	<0.001	680	96	16.46(12.29–21.69)	12.87(10.06–16.32)	0.200
Livelihood efforts (> = more)		676	16.44(15.85–17.04)	17.17(16.67–17.68)	0.067		680	15.31(14.69–15.93)	15.61(15.14–16.08)	0.453
Stress about lack of work work (> = more)		676	12.20(11.84–12.55)	12.04(11.74–12.33)	0.494		680	12.08(11.69–12.47)	12.04(11.77–12.31)	0.862
Feelings of shame about lack of work (> = more)		676	10.86(10.57–11.16)	11.00(10.75–11.25)	0.496		680	10.72(10.39–11.05)	11.07(10.83–11.30)	0.091
Stolen in past 4 weeks as hungry: Yes	676	249	25.52(20.81–30.88)	45.57(40.63–50.61)	<0.001	680	171	21.94(17.09–27.70)	26.86(22.95–31.17)	0.161
Borrowed past 4 weeks (weekly or more)	676	244	31.03(25.99–36.57)	39.84(35.08–44.81)	0.018	680	215	25.74(20.62–31.63)	34.76(30.46–39.33)	0.015
**Gender attitudes/relationships**										
Gender attitudes (> = less equitable)		675	26.65(25.52–27.78)	29.01(28.09–29.92)	0.002		680	23.78(22.58–24.98)	26.52(25.64–27.39)	<0.001
Controlling behaviours (> = more)		674	10.09(9.64–10.53)	11.53(11.16–11.89)	<0.001		680	8.41(7.89–8.92)	11.30(10.91–11.69)	<0.001
Childhood traumas (> = more)		675	5.52(4.90–6.14)	8.65(7.99–9.30)	<0.001		680	4.99(4.36–5.63)	7.29(6.75–7.82)	<0.001
Quarrelling in relationship (> = more)		675	0.51(0.45–0.58)	0.74(0.69–0.81)	<0.001		680	0.55(0.46–0.64)	0.87(0.80–0.94)	<0.001
**Mental Health**										
Alcohol use (> = more)		677	5.56(4.78–6.34)	9.55(8.68–10.43)	<0.001		680	2.40(1.86–2.95)	5.31(4.65–5.98)	<0.001
Drug use past 12m: Yes	670	348	40.77(35.19–46.59)	60.31(55.29–65.13)	<0.001	680	216	18.57(14.12–24.02)	38.83(34.37–43.47)	<0.001
Hope (> = more)		669	13.91(13.43–14.40)	12.38(11.92–12.83)	<0.001		680	13.72(13.16–14.27	13.35(12.94–13.77)	0.300
Views life (> = more positive)		676	9.97(9.55–10.39)	10.50(10.15–10.85)	0.059		680	9.88(9.43–10.34)	9.91(9.58–10.24)	0.923
Life success (> = more successful)		676	2.02(1.88–2.17)	2.35(2.21–2.50)	0.002		680	2.46(2.26–2.65)	2.50(2.35–2.65)	0.703
Depression (> = more depressed)		671	23.56(22.44–24.69)	26.65(25.63–27.68)	<0.001		680	23.38(22.05–24.71)	27.20(26.18–28.22)	<0.001
PTSD (yes)	677	103	11.69(10.54–12.83)	14.55(13.57–15.54)	<0.001	680	144	11.49(10.14–12.84)	16.16(15.19–17.12)	<0.001

In terms of poverty and IPV, for men there were ambiguous trends in the data. For men, two work related variables, earning any money in the past month (p<0.001) and working in the past 12 months (p<0.001), were associated with a greater proportion of men reporting perpetrating past year IPV, compared to men who did not. While, two measures of individual livelihood strategies, were also associated with a higher proportion of men reporting past 12 month IPV perpetration, specifically stealing in the past four weeks because of hunger (p<0.001) and borrowing food or money because of hunger (p = 0.018), compared to those who did not.

For women, household food insecurity was associated with an increase in IPV experienced, whereby those reporting the greatest household food insecurity were more likely to experience IPV, compared to those who experienced no or little household food insecurity (p<0.001). In addition, a greater proportion of women reporting borrowing more frequently because of hunger were more likely to report experiencing IPV (p = 0.0152), compared to those who reported no IPV.

For men and women gender equity and relationship measures were strongly associated with IPV perpetration and experience respectively. Men reporting perpetrating IPV and women experiencing IPV, were more likely to report less gender equitable attitudes (p = 0.002 men; p<0.001 women), higher levels of controlling behaviours (p<0.001 men; p<0.001 women), more childhood traumas (p<0.001 men, p<0.001 women) and arguing more in their relationship (p<0.001 men, p<0.001 women), compared to those not perpetrating (men) and experiencing (women) IPV.

All measures of poorer mental health and higher levels of substance use were also associated with increased IPV perpetration for men and experience for women. Men reporting IPV perpetration and women reporting IPV experience reported higher mean scores for alcohol use (p<0.001 men; p<0.001 women), and a greater proportion reported past year drug use (p<0.001 men; p<0.001 women), compared to those who did not. Men perpetrating IPV and women experiencing IPV reported higher mean scores for depressive symptoms (p<0.001 men; p<0.001 women), and a larger proportion reported PTSD symptoms (p<0.001 men; p<0.001 women), compared to those who did not. Men reporting past year IPV perpetration had lower mean hope scores (p<0.001), higher views on life scores (p = 0.059) and higher life success scores (p = 0.002), compared to men who did not perpetrate IPV.

Men who reported higher levels of ‘hope’ were less likely to perpetrate IPV (p<0.001). Finally, men who reported greater success in life, compared to other men, were more likely to report IPV perpetration (p = 0.002).

In the adjusted logistic regression model (Table 3) for men, not having a current partner was associated with reduced IPV perpetration (aOR0.37, p = 0.006), compared to those living with a current partner. Following bivariate analysis, measures of individual work, specifically any past month earnings, compared to those who reported zero past month earnings (a1.46, p = 0.041) and working consistently in the past year, compared to those reporting a lack of consistency (aOR1.74, p = 0.013) were associated with more IPV perpetration. Men who had stolen in the past month (compared to those who did not steal) were also more likely to report IPV perpetration (aOR1.75, p = 0.004). IPV perpetration was also associated with men reporting more controlling behaviours (aOR1.07, p<0.009), more childhood traumas (aOR1.05, p<0.002), and more quarrelling in a relationship (aOR1.49, p<0.01). In terms of substance use, greater alcohol (aOR1.04, p<0.003) and past year drug use (compared to no use) (aOR1.47 p = 0.040) were associated with more IPV perpetration. Being more hopeful was associated with reduced IPV perpetration (aOR0.93, p = 0.001), while perceptions of greater life success was associated with increased IPV perpetration (aOR1.19, p = 0.013).

**Table 3:** Multivariable model showing adjusted odds ratios for factors associated with men's perpetration, and women's experience, of past 12 month physical and/or sexual IPV.

**Men** ^a^^,^^b^	**aOR**	**95%CI**	**p-value**
Relationship status			
Living with partner	ref		
Partner, but not living with	0.74	0.39–1.39	0.352
No current partner	0.37	0.18–0.75	0.006
Earnt any money in past month (> = R1; yes)	1.46	1.02–2.09	0.041
Worked in the past 12months (most or each month c.f. less)	1.74	1.12–2.69	0.013
Stolen in past 4 weeks as hungry: Yes (c.f. once or more)	1.75	1.20–2.55	0.004
Controlling behaviours (> = more)	1.07	1.02–1.12	0.009
Quarrelling in relationship (> = more)	1.49	1.12–1.98	0.007
Alcohol use (> = more)	1.04	1.01–1.07	0.003
Drug use past 12m: Yes (c.f no)	1.48	1.01–1.08	0.040
Childhood traumas (> = more)	1.05	1.02–1.09	0.002
Hope (> = more)	0.93	0.90–0.97	0.001
Life success (> = more successful)	1.18	1.04–1.35	0.013
**Women** ^a^^,^^c^	**aOR**	**95%CI**	**p-value**
Relationship status			
Living with partner	ref		
Partner, but not living with	0.51	0.30–0.87	0.013
No current partner	0.71	0.37–1.34	0.290
Household food insecurity			
None or little	ref		
Moderate	1.48	0.93–2.38	0.100
High	1.84	1.08–3.14	0.025
Controlling behaviours (> = more)	1.13	1.08–1.18	<0.0001
Quarrelling in relationship	1.57	1.22–2.03	<0.0001
Alcohol use (> = more)	1.07	1.02–1.11	0.002
Drug use past 12m: Yes (c.f no)	1.88	1.20–2.95	0.002
Depression (> = more depressed)	1.02	1.00–1.04	0.034

aOR adjusted odds ratios; 95%CI 95 percent confidence intervals

^a^ Controlling for age, education, intervention arm and adjusted for clustering

^b^ n = 668, p<0.0001

^c^ n = 680, p<0.0001

For women (Table 3), the multivariable logistic regression model showed that women who had a partner, but did not live with them, were less likely to experience IPV (aOR0.53, p<0.05), compared to women who lived with their partners. Household food insecurity was associated with more IPV experience, whereby those reporting the highest levels of food insecurity were more likely to report IPV experience, compared to those reporting no or little food-insecurity (aOR1.84 p = 0.025). IPV experience was associated with more controlling behaviours (aOR1.14, p<0.0001), and more quarrelling in the relationship (aOR1.57, <0.0001). Women reporting IPV experience reported more alcohol use (aOR1.06, p = 0.002) and drug use (compared to none) (aOR1.75, p = 0.0025). Women reporting IPV was associated with higher depression scores (aOR1.02, p = 0.034).

**Fig 2:**
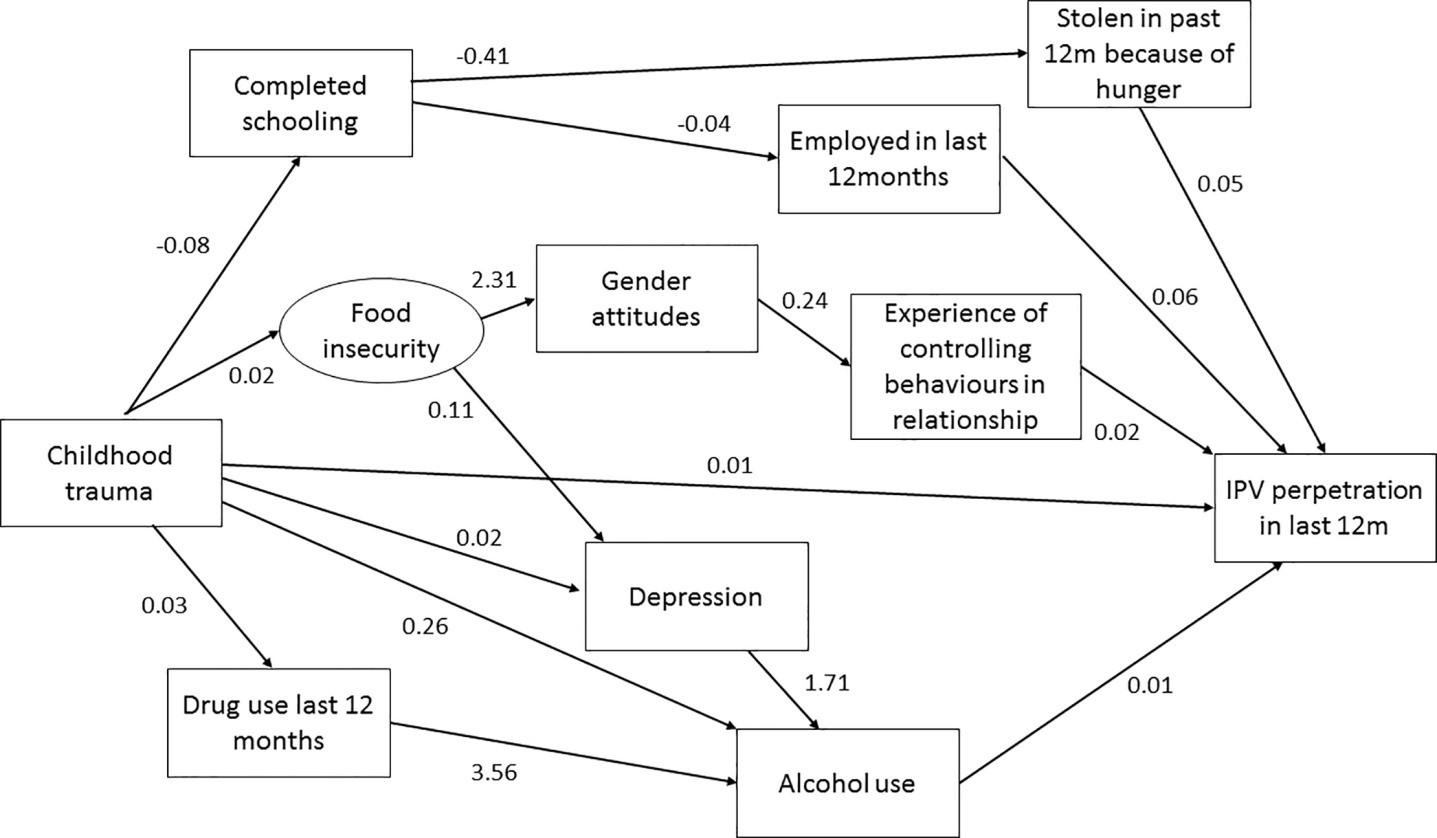
Men’s structural equation model showing direct and mediated pathways between childhood traumas and IPV perpetration.

In the men’s SEM (Fig 2; Tables 4 & 5) there was a direct pathway between childhood traumas and IPV perpetration, whereby the coefficient indicated that higher childhood trauma scores were associated with increased IPV perpetration. A set of pathways mediated the relationship between childhood traumas and IPV perpetration, through substance use and mental health. Alcohol use was directly associated with increased IPV, and there was a direct pathway from childhood trauma to alcohol use and a mediated pathway from childhood trauma to drug use and to alcohol use. A second set of mediated pathways were linked to alcohol use, specifically a pathway from childhood trauma to household food insecurity and then depression and another from childhood trauma directly to depression. There was a direct pathway from depression to alcohol use.

**Table 4:** Treatment of variables in the structural equation model.

			Women	Men
Observed Variables	Indicator	Definition	R2	R2
Any IPV past 12 months perpetration men/ experience women	Dichotomous	8 items asking about experiences of physical and sexual IPV, scored never, once, few, many. Recoded into dichotomous yes/no. Based on WHO VAW scale.	0.15	0.24
Educational Attainment			0.07	5.51
Gender Attitudes	Continuous	20 items asking about attitudes, scored 1–4 and summed. Example: A woman should tolerate violence in order to keep her family together, based on the GEMS scale	0.17	93.25
Drug use past year	Continuous	Single item: In the last 12 months how many times have you used drugs to make you high or have a good time? Responses, never, once, many	0.01	0.62
Childhood trauma	Continuous	12 items asking about physical, emotional, sexual abuse and neglect and food insecurity before age of 18. Scored 1–4 and summed.	0.04	
Controlling behaviours	Continuous	8 items, scored from 1–5 (strongly disagree—strongly agree) and summed. When I wears things to make me look beautiful he thinks I may be trying to attract other men	-0.06	14.91
Arguing	Continuous	One item asking about frequency of arguing in relationship, scored 1–3, rarely, sometimes, often	0.09	
Alcohol use	Continuous	9 items forming AUDIT scale, with range of scores 0–40	-0.14	
Depressive symptoms	Dichotomous	20 items asking about past week symptoms of depression, each item scored 0–3, and dichotomized at 20/21	0.10	0.11
Stealing for lack of money	Continuous	One item asking about stealing in past month for lack of money, scored 1–4		0.96
Employment in last 12 months	Continuous	One item asking about employment in past 12 months, scored 1–4		0.87
**Overall**			**0.85**	**0.237**

**Table 5:** Structural equation model for men.

Parameter	Standardized coefficients	SE	z	P>|z|	95% Conf. Interval	
Direct effects						
Poverty → depression	0.111	0.038	2.96	0.003	0.037	0.185
Childhood trauma →depression	0.021	0.003	8.14	<0.0001	0.016	0.026
School completion → gender attitudes	-1.260	0.150	-8.4	<0.0001	-1.550	-0.960
Poverty → gender attitudes	2.310	0.778	2.970	0.003	1.550	0.960
Depression → Problem drinking	1.710	0.582	3	0.003	0.592	2.838
Drug use→ Problem drinking	3.558	0.359	9.91	< .0001	2.853	4.263
Childhood trauma→ Problem drinking	0.239	0.062	4.2	< .0001	0.138	0.380
Childhood trauma → Drug use	0.026	0.005	5.01	<0.0001	0.016	0.036
Childhood trauma → School completion	-0.075	0.013	-5.46	<0.0001	-0.102	-0.048
School completion → Stealing due to lack of food	-0.407	0.089	-4.58	<0.0001	-0.582	-0.232
School completion → Employment in the last 12m	-0.040	0.016	-2.5	0.013	-0.072	-0.009
Gender attitudes → Control in the relationship	0.237	0.014	16.81	< .0001	0.210	0.265
Childhood trauma → Poverty	0.024	0.004	5.61	< .0001	0.016	0.033
Problem drinking → IPV	0.010	0.002	4.89	< .0001	0.006	0.015
Stealing for lack of money for food → IPV	0.053	0.018	2.94	0.003	0.018	0.089
Employment in the last 12 m→ IPV	0.056	0.019	3.00	0.003	0.019	0.092
Relationship control → IPV	0.016	0.005	3.36	0.001	0.007	0.026
Childhood trauma → IPV	0.011	0.003	3.75	<0.0001	0.005	0.017

Two mediated pathways linked childhood trauma, schooling and IPV for men. Childhood traumas decreased educational attainment. A mediated pathway to IPV was through stealing in the past month because of hunger and this was inversely associated with completion of secondary schooling and positively associated with IPV perpetration. A second mediated pathway showed those with more schooling were less likely to have been employed in the past 12 months, but those who had been employed were more likely to perpetrate IPV.

For men, a final set of pathways between childhood trauma and IPV perpetration were mediated by gender attitudes and controlling behaviours. Childhood traumas increased current household food insecurity, which in turn was associated with less gender equitable attitudes. A second pathway to gender attitudes was through schooling whereby those with more schooling had more equitable gender attitudes. The pathway from gender attitudes to IPV perpetration was mediated by controlling behaviours.

**Fig 3:**
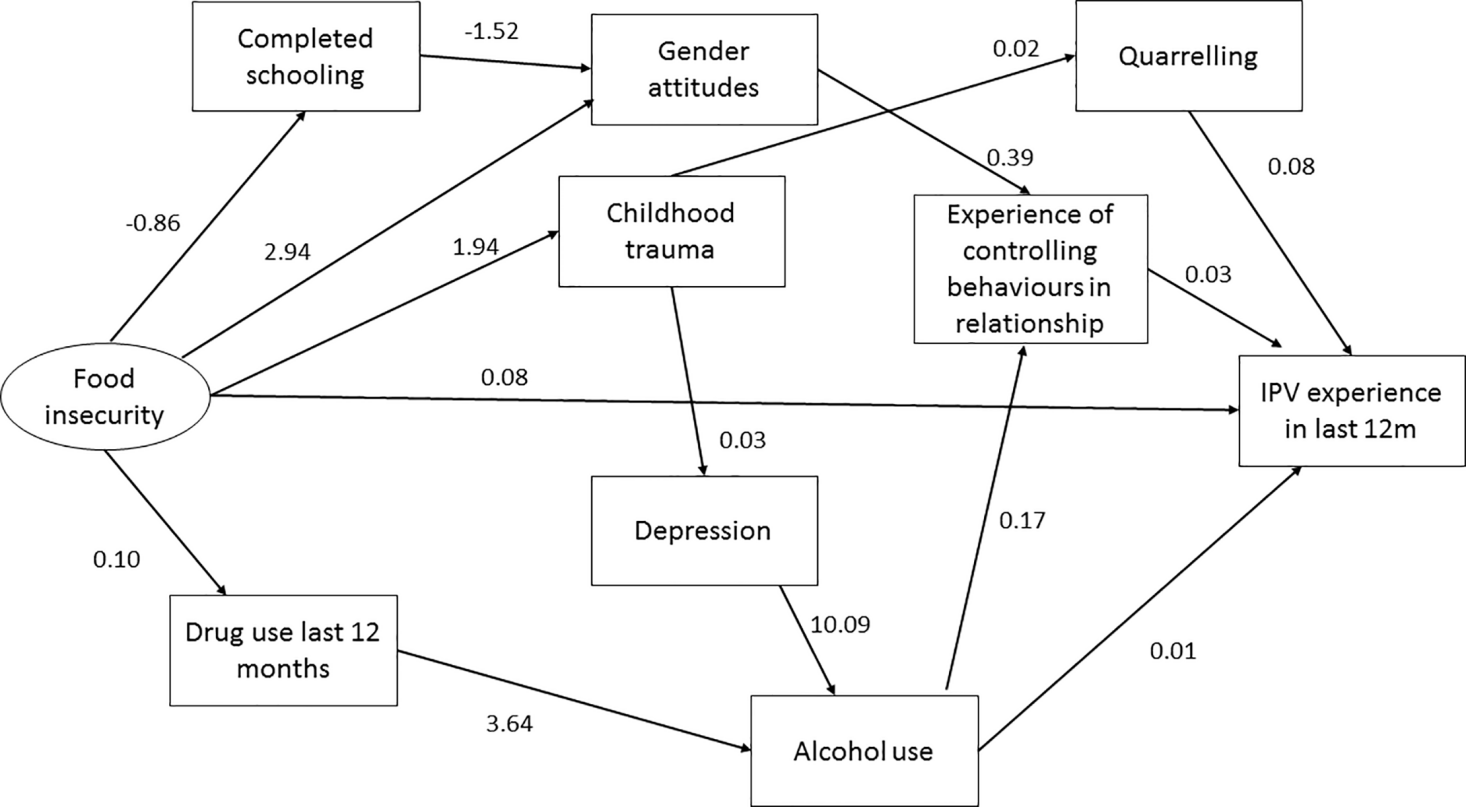
Structural equation model showing direct and mediated pathways between poverty and women’s experiences of intimate partner violence.

For women (Fig 3; Tables 4 & 6) the SEM showed a direct pathway between food insecurity and IPV experienced, with the coefficient indicating that women who had higher levels of food insecurity experienced greater levels of IPV. There were a number of mediated pathways between poverty and IPV. One mediated pathway was between poverty and substance use and mental health. Alcohol use was directly associated with increased IPV experience. There was no direct pathway between food insecurity and alcohol use, however there was a pathway mediated by drug use and a second pathway from food insecurity to childhood trauma and then depression to alcohol use.

**Table 6:** Structural equation model for women.

Parameter	Standardized coefficients	SE	z	P>|z|	95% Conf. Interval
Direct effects						
Relationship control → IPV	0.023	0.004	6.00	<0.0001	0.017	0.033
Arguing → IPV	0.083	0.023	3.60	<0.0001	0.038	0.129
Alcohol → IPV	0.012	0.002	5.52	<0.0001	0.008	0.016
Poverty → IPV	0.076	0.033	2.29	0.022	0.011	0.141
Poverty→ Education	-0.863	0.156	-5.52	<0.0001	-1.171	-0.556
Education → Gender attitudes	-1.515	0.172	-8.80	<0.0001	-1.853	-1.177
Poverty → Gender attitudes	2.939	0.674	4.36	<0.0001	1.616	4.261
Poverty → Drug use	0.097	0.045	2.17	0.03	0.009	0.185
Poverty → Childhood trauma	1.943	0.444	4.38	<0.0001	1.071	2.815
Gender attitudes → Relationship control	0.388	0.073	5.31	<0.0001	1.071	2.815
Alcohol → Relationship control	0.171	0.051	3.37	0.001	0.071	0.270
Childhood trauma → Arguing	0.018	0.005	3.35	0.001	0.007	0.028
Relationship control → Arguing	0.038	0.007	5.74	<0.0001	0.025	0.511
Drugs → Alcohol	3.646	0.392	9.31	<0.0001	2.877	4.416
Depression → Alcohol	10.087	2.306	4.37	<0.0001	5.559	14.614
Childhood trauma → Depression	0.027	0.003	10.39	<0.0001	0.022	0.032

Another set of pathways between household food insecurity and IPV were mediated by intimate relationships. Household food insecurity reduced educational attainment, and lower educational attainment was associated with less gender equitable attitudes. There was also a direct pathway from food insecurity to gender attitudes, where women with higher levels of food insecurity had less gender equitable attitudes. Gender attitudes were mediated by their experience of controlling behaviours from men to IPV experience. Another mediated pathway was between childhood trauma and quarrelling in relationships. Childhood traumas increased quarrelling and quarrelling was associated with IPV experience. Controlling behaviours also increased quarrelling in the model. In both models the goodness of fit statistics were satisfactory.

## Discussion

The analysis presented here shows that for women and men the relationship between poverty and IPV experience for women and perpetration for men in this sample is complex. However, this analysis clearly highlights four major themes. First, a range of markers of poverty intersect with gender identity to shape IPV perpetration or experience. Second, the study reinforces the importance of gender inequalities in driving IPV. Third, the models help clarify the relationship between poor mental health and substance use, and IPV experience or perpetration. Finally, the models also highlight a resilience pathway that is protective of IPV for women and men.

As with global literature [4–6]household food insecurity was associated with increased IPV experience for women. Specifically, in the adjusted regression model, women reporting the highest levels of household food insecurity, and who had borrowed in the past four weeks because of hunger, were significantly more likely to experience IPV. In addition there was a direct pathway between household food insecurity and IPV experience in the SEM. This reflects global research that highlights the importance of household food insecurity in women’s experience of IPV [5, 6]. Importantly, the SEM showed how food insecurity shaped women’s subsequent experiences of education, childhood trauma, and depression, demonstrating how widespread inequalities structure themselves at an early age undermining women’s long-term development [6]. The focus on food insecurity for women, as opposed to other forms of markers of poverty, fits into a wider argument about how women in particular experience greater impacts from food insecurity, including in terms of depression, [43] than men. There are two potential reasons for the association between food insecurity and IPV experience amongst women. First, food insecurity at the household level may increase stress in relationships, with stress leading to the use of violence in relationships [8]. Second, food insecurity may be a marker of women’s economic dependency, and this economic dependency leaves women unable to leave violent relationships [2]. It may explain why some studies have shown that direct cash transfers to women in the form of social protection interventions, can reduce women’s experiences of IPV, and this may be through reducing the stress of food insecurity, and enabling women to obtain some economic dependency [8, 44].

For men, the SEM and regression model, help untangle the ambiguous set of research findings about the role of poverty in shaping IPV perpetration, through emphasizing how expectations of male economic provision is a key driver of IPV perpetration. In the regression model and the SEM, in contrast to the women’s models, factors associated with IPV perpetration were strongly associated with economic provision, and providing in relationships, rather than necessarily absolute experiences of food insecurity. In the regression model, food insecurity was not associated with IPV, and there was no direct pathway between food insecurity and IPV perpetration in the SEM. Rather, pathways which were more direct, were associated with attempts at economic provision, whether through work, or through stealing as a way to provide. This argument reinforces analyses of rape perpetration in South Africa, which has suggested that men who rape are slightly better off socially and economically, although living in overall contexts of poverty [20]. This argument suggests that through economic provision in relationships, men develop a sense of sexual entitlement, that lead to male perpetration of IPV. This connects with the finding in the regression model that men who reported greater feelings of ‘life success’ were more likely to perpetrate IPV.

The models also show clearly, for women and men, the central role played by gender power in driving IPV, in the form of gender attitudes and male controlling behaviours. In both SEMs, greater levels of food insecurity, increased gender inequitable attitudes, which, via controlling behaviours, increased IPV perpetration (men) and experience (women). The central role of gender inequalities and male control over women in IPV perpetration and experience, has been recognized widely previously in literature [6, 15], but these models emphasise the importance of food insecurity in shaping these associations. This has important implications for interventions that seek to use economic strengthening approaches to reduce IPV, highlighting that economic strengthening interventions for women, and for men, are unlikely to successfully prevent IPV, unless they are combined with interventions that also seek to transform dominant gender relationships and strengthen women’s social power [45, 46].

There were also similarities in the role mental health played in driving IPV for women and men. In both regression models, IPV was strongly associated with substance use, and a range of measures of poor mental health indicators. The SEMs showed that food insecurity and childhood traumas were associated with poorer mental health, and more substance use, and that these in turn were associated with IPV perpetration (men) or experience (women). This reinforces the multiple impacts the food insecurity has in shaping IPV both directly (for women) and indirectly, and emphasizes the structural nature of food insecurity. It also supports global literature that increasingly recognizes the role of poor mental health and substance abuse in driving IPV perpetration or experience, and not only viewing these as an outcome of IPV [18, 27].

Importantly in both SEMs there was a resilience pathway, through education, and gender attitudes that reduced IPV. Women and men with more education had more gender equitable attitudes, which reduced controlling behaviours, and therefore IPV. Studies have suggested that education is protective of IPV for women, although the exact pathways through which this occurs is unclear, and other studies have suggested education is a risk factor for IPV [9]. This resilience pathway has been described previously for women [6], but not for men. As education outcomes were associated with food insecurity for women, and childhood trauma for men, the importance of tackling these structural forms of inequalities early in life remain crucial, in shaping educational outcomes and in turn IPV.

The study has a number of limitations. First, we did not ask about all possible markers of poverty, for instance assets, rather we only asked about a select few. This was based on discussions about what may be most relevant for this population. Second, there are issues about temporal sequencing of events as data is cross-sectional, though this is somewhat overcome through the use of the SEM. Third, we made an assumption that food-insecurity was unlikely to have changed over time, and so was modelled to the left of the SEM, and thus a key pathway to historical experiences (such as education and childhood traumas). We did this following other models of recent IPV experience [6]. Third, our population self-selected to participate in an intervention trial, and as such the findings are not generalizable the population of informal settlements, with likely an over-representation of those unemployed, however this does not necessarily invalidate the associations seen in the data. Finally, there was not much variation amongst the cohort, given the inclusion/exclusion criteria, for many of the measures, and as such this reflects findings from one very specific group.

## Conclusions

This group of young women and men living in urban informal settlements experience and perpetrate (respectively) exceedingly high levels of IPV. While studies tend to focus on individual level attributes for risk and protective factors, this analysis has clearly shown the importance of structural drivers of IPV in this population, namely food insecurity, which has multiple impacts on young people’s lives, in turn increasing vulnerability or perpetration of IPV. Moreover, the analysis highlighted the importance of considering different forms of poverty in shaping IPV dynamics, rather than just using a ‘blanket term’ of poverty.

Importantly the analysis highlights a number of modifiable risk factors that may be amenable to intervention. Clearly ‘poverty’ reduction, in the form of reducing food insecurity is critical for the promotion of more equitable societies and the reduction of IPV, but interventions to achieve this need to be combined with interventions that focus on supporting women and men to rethink gender relationships, if impact is to be maximized [26, 45]. Similarly, reducing childhood traumas, including sexual and physical abuse, remains a priority for effective IPV prevention. For women and men it was evident that schooling is protective of IPV, and that the promotion of education could have long-term benefits. Finally, the centrality for women and men, of pathways from food insecurity to poor mental health and substance abuse, highlight the importance of working to promote mental health amongst young people in informal settlements, but attempts to do this need to be tied to interventions that work to reduce underlying food insecurity, which were driving poor mental health in this population.

## Supporting information

S1 Data Men’s data for analysis. (DTA)

S2 Data Women’s data for analysis. (DTA)
